# Microsurgical Clipping of Unruptured Anterior Communicating Artery Aneurysm—A Single-Center Experience

**DOI:** 10.3390/brainsci15121272

**Published:** 2025-11-27

**Authors:** Nico Stroh-Holly, Stefan Aspalter, Milan Slavomir Vosko, Benjamin Laudien, Philipp Hermann, Helga Wagner, Michael Sonnberger, Maria Gollwitzer, Philip Rauch, Wolfgang Senker, Andreas Gruber, Matthias Gmeiner

**Affiliations:** 1 Department of Neurosurgery, Kepler University Hospital, Johannes Kepler University Linz, 4040 Linz, Austria; nico.stroh-holly@kepleruniklinikum.at (N.S.-H.);; 2 Center for Clinical Studies (CCS Linz), Johannes Kepler University Linz, 4040 Linz, Austria; 3 Institute of Applied Statistics, Johannes Kepler University Linz, 4040 Linz, Austria; 4 Institute of Neuroradiology, Kepler University Hospital, Johannes Kepler University Linz, 4040 Linz, Austria; 5 Clinical Research Institute for Neuroscience, Johannes Kepler University Linz, 4040 Linz, Austria

**Keywords:** intracranial aneurysm, anterior communicating artery aneurysm, clipping, microsurgical treatment, outcome

## Abstract

**Background/Objectives**: Unruptured aneurysms of the anterior communicating artery (AComA) are associated with a higher risk of rupture and present unique anatomical challenges. Although endovascular techniques have advanced considerably, microsurgical clipping continues to represent an essential treatment option, particularly for complex cases. We conducted a retrospective analysis to evaluate outcomes of microsurgical clipping for unruptured AComA aneurysms over an 18-year period at a high-volume tertiary neurosurgical center. **Methods**: A retrospective analysis was conducted on 106 patients who underwent microsurgical clipping for unruptured AComA aneurysms between 2002 and 2020. Preoperative, intraoperative, and postoperative parameters were assessed. Excluded were previously ruptured or previously surgically treated aneurysms. Logistic regression models were used to identify predictors of postoperative complications with a focus on aneurysm projection. **Results**: Complete angiographic occlusion was achieved in 92.2% of cases, with a retreatment rate of 0.9%, which is comparable to the recent literature. Permanent neurological deficits occurred in 5.7% of patients. Posterior aneurysm projection was significantly associated with postoperative infarction and permanent neurological deficits. Intraoperative ICG angiography was associated with a reduced risk of ischemic complications. **Conclusions**: Microsurgical clipping remains a safe and effective treatment for unruptured AComA aneurysms in selected patients, offering durable occlusion and low complication rates. Aneurysm projection is a key predictor of outcome, and intraoperative ICG angiography significantly enhances surgical safety. These findings support the continued role of microsurgery in the interdisciplinary management of AComA aneurysms.

## 1. Introduction

Unruptured intracranial aneurysms (UIAs) are increasingly detected due to the widespread use and improved resolution of non-invasive neuroimaging techniques over the last few years, with a reported prevalence of approximately 3% [1]. Aneurysms of the anterior communicating artery (AComA) represent 25–30% of all intracranial aneurysms [2,3,4,5].

Several studies have shown that the natural history of AComA aneurysms is characterized by a higher risk of rupture compared to other aneurysms [6,7,8]. The presence of risk factors further increases the rupture risk [9,10,11]. Rupture of an AComA aneurysm typically results in subarachnoid hemorrhage (SAH), associated with high morbidity and mortality [12].

The decision regarding treatment of an unruptured AComA aneurysm continues to be challenging and should be individualized according to patient characteristics and aneurysm morphology [13]. A comprehensive evaluation is required to determine whether an aneurysm can be considered stable or is at risk of rupture. The likelihood of rupture is multifactorial and depends not only on clinical parameters [14,15] but also on lifestyle-related risk factors [16,17]. In addition, several studies have shown that the morphological characteristics of aneurysms [18,19] and hemodynamic parameters [20] serve as important indicators of rupture risk and should be taken into account [21].

Balancing the risk of rupture against potential treatment complications is central to decision-making for AComA aneurysms. This risk-benefit assessment directly influences the choice between available treatment strategies, which include microsurgical clipping and various endovascular approaches [22]. Until the 1990s, microsurgical clipping was the only available treatment modality for AComA aneurysms [23]. With the introduction of Guglielmi detachable coils (GDCs), endovascular techniques have gained increasing attention and have also become an essential part of aneurysm management [24]. Since then, various endovascular treatment strategies have emerged, including coiling with balloon or stent assistance, as well as the implantation of Flow Diverters or intrasaccular devices [25,26,27].

Nevertheless, microsurgical clipping remains the treatment modality of choice for many AComA aneurysms, particularly in cases involving complex vascular anatomy, wide necks, or unfavorable dome-to-neck ratios [28]. The AComA region is anatomically complex, characterized by the presence of highly eloquent perforating arteries, especially the recurrent artery of Heubner [29]. Frequent anatomical variants such as hypoplastic A1 segments pose significant challenges for both endovascular and microsurgical approaches [30].

**Figure 1:**
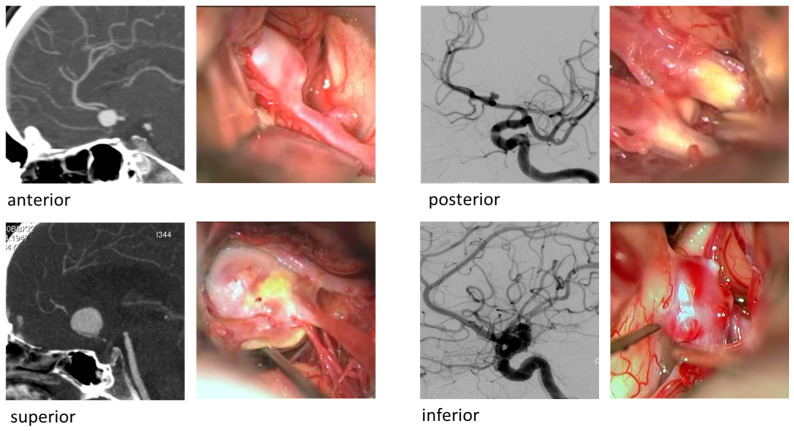
Illustration of representative cases of the cohort—Anterior communicating artery aneurysms and the four aneurysm projections: anterior, posterior, superior and inferior.

Studies have shown that the projection of the aneurysm dome should influence the choice of treatment modality [31,32]. AComA aneurysms may project in various directions—anteriorly, superiorly, inferiorly, or posteriorly (Figure 1)—resulting in different risk profiles depending on their proximity to perforating arteries and the hypothalamic region [33].

Despite the growing body of literature on microsurgical and endovascular treatment of AComA aneurysms, robust long-term outcome data, particularly for microsurgical clipping in unruptured cases, remain scarce. This evidence gap complicates treatment decisions, especially in anatomically complex cases where microsurgery may still be preferred. To address this gap and to provide comprehensive data to the ongoing discussion regarding the optimal management of AComA aneurysms and the continued role of microsurgical clipping in selected cases, we present an 18-year single-center analysis of clinical and radiological outcomes following microsurgical clipping of unruptured AComA aneurysms at a high-volume neurosurgical department.

## 2. Materials and Methods

The retrospective single-center study was conducted at the Department of Neurosurgery, Kepler University Hospital, Linz, Austria, and ethical approval was obtained from the local Ethics Committee of the Federal State of Upper Austria (EK-No.: 1318/2020).

### 2.1. Patient Selection

All Patients treated for an unruptured AComA aneurysm with microsurgical clipping between January 2002 and December 2020 at the neurosurgical department of the Kepler University Hospital in Linz, Austria were retrospectively identified and systematically entered into a database for subsequent analysis.

Inclusion required the confirmation of an AComA aneurysm by preoperative neuroradiological imaging, such as digital subtraction angiography (DSA) or computed tomography angiography (CTA). Microsurgical clipping had to be recommended as the primary treatment modality by an interdisciplinary cerebrovascular board comprising both endovascular and microsurgical specialists.

Patients were excluded if the aneurysm was previously ruptured or had prior surgical intervention on the same AComA aneurysm. The presence of a coincidental additional aneurysm was not considered an exclusion criterion.

A board-certified neuroradiologist retrospectively reviewed radiological imaging data. In cases of incomplete initial reports, missing findings were subsequently supplemented by the same neuroradiologist.

### 2.2. Preoperative Parameter

Preoperative data were categorized into patient-specific and aneurysm-specific parameters. Patient-specific variables included sex, age (in years), American Society of Anesthesiologists (ASA) score (ranging from 1 to 5, as a standardized measure of preoperative physical status and comorbidity burden), and relevant comorbidities such as autosomal dominant polycystic kidney disease (ADPKD), history of subarachnoid hemorrhage (SAH), arterial hypertension, diabetes mellitus, smoking status, and alcohol abuse—parameters that impact rupture risk assessment and may affect aneurysm morphology and treatment complexity.

Aneurysm-specific parameters included the projection of the aneurysm fundus, which was assessed using DSA or CTA. Fundus projection was categorized as anterior, superior, inferior, or posterior based on the projection axis. In addition, aneurysm-specific variables comprised the maximum diameter (in millimeters), prior endovascular coiling, presence of intraluminal thrombosis, aneurysm morphology, calcifications, and the number of coincidental aneurysms.

### 2.3. Surgical Technique and Intraoperative Monitoring

Microsurgical clipping was performed via a frontotemporal/pterional approach in all cases. Surgical planning included assessment of the dominant inflow vessel to maintain physiological flow patterns, guided by preoperative angiography and intraoperative ICG angiography. All procedures were carried out by board-certified neurosurgeons, specialized in cerebrovascular microneurosurgery.

The following intraoperative parameters were systematically documented: side of surgical approach, number of clips applied, necessity for clip repositioning, and occurrence of intraoperative aneurysm rupture. Temporary clipping of the A1 segment of the anterior cerebral artery was employed when required to achieve proximal vascular control.

Additional monitoring parameters were also recorded, including the use of intraoperative neuromonitoring with somatosensory evoked potentials (SSEP) and motor evoked potentials (MEP) to assess neurological integrity, as well as the application of indocyanine green (ICG) videoangiography to verify clip positioning and vessel patency.

Both the subjective intraoperative aneurysm occlusion rate and the radiologically verified occlusion rate were recorded, based on either intraoperative DSA or immediate postoperative DSA.

### 2.4. Outcome Parameters

Postoperative aneurysm occlusion was evaluated using DSA, either intraoperatively or within the first 24 h postoperatively. The degree of occlusion was classified according to the Raymond–Roy Occlusion Classification (RROC), distinguishing between complete occlusion (Class I), residual neck (Class II), and residual aneurysm (Class III).

In addition, a comprehensive set of postoperative clinical and radiological parameters was assessed: occurrence of postoperative intracerebral hemorrhage (ICH), remote cerebellar hemorrhage (RCH), chronic subdural hematoma (cSDH), and the need for revision surgery. Routine postoperative CT imaging was performed to exclude hematoma or significant brain edema. Ischemic complications such as cerebral infarction were documented, as well as new neurological deficits, which were further differentiated into transient and permanent deficits (defined as persisting beyond four weeks postoperatively). Other recorded complications comprised postoperative seizures, surgical site infections (defined as any wound infection requiring treatment, either antibiotic therapy or surgical intervention), and perioperative mortality.

Furthermore, long-term outcome parameters included the rate of aneurysm recurrence and the necessity for retreatment.

### 2.5. Statistical Analysis

The statistical analysis was conducted at the Center for Clinical Studies (CCS), Johannes Kepler University in Linz. Statistical analysis included descriptive statistics for all valid observations. For nominal variables, absolute and relative frequencies, and for metric variables, mean and standard deviation (SD), as well as median and range, were computed. Logistic regression models were applied to evaluate the association of preoperative and intraoperative parameters with postoperative outcomes. Variable selection was performed by stepwise forward and backward procedures. In case of quasi-complete separation, penalized likelihood estimation was applied to obtain stable regression estimates.

All statistical analyses were performed using the statistical software R Version 4.5.1 (R Foundation for Statistical Computing, Vienna, Austria).

## 3. Results

In this retrospective monocentric study, 106 patients undergoing surgical treatment for AComA aneurysms were included. The mean age was 52.9 years, and 11.3% had a documented history of SAH. Perioperative risk was assessed using the American Society of Anesthesiologists (ASA) physical status at admission in 80 patients, with the majority of patients (n = 47, 58.8%) classified as ASA 2 and only one patient presenting with an ASA score above 3. Baseline patient characteristics are summarized in Table 1.

Given the relevance of aneurysm projection to surgical approach and outcome, the distribution of projections was examined in detail. The most frequent projection was anterior (n = 50, 47.2%), followed by superior (n = 32, 30.2%), inferior (n = 14, 13.2%), and posterior projection (n = 10, 9.4%). The mean maximal diameter was 5.9 mm (range, 1–19 mm). Seven aneurysms (6.6%) had been previously treated with coiling but required surgical clipping due to recurrence.

Preoperative imaging was performed predominantly via DSA in 91 (85.9%), CTA in 13 (12.3%) and MRA in 2 (1.9%) patients. Bleb formation, defined as focal outpouching of the aneurysm wall suggestive of localized wall weakness, was detected in preoperative imaging in 60 cases (56.6%). Detailed aneurysm-specific parameters are summarized in Table 1.

### 3.1. Intraoperative Parameters

The average number of clips applied per aneurysm was 1.3 (range 0–3). Clip repositioning was required in 21 procedures (19.8%), whereas intraoperative rupture occurred in 3 cases (2.8%). Temporary clipping of the parent artery was performed in 12 cases (11.3%). Intraoperative neuromonitoring, including MEP and SSEP, was utilized in 31 interventions (29.3%), and intraoperative ICG angiography was conducted in 41 procedures (38.7%). The surgical approach was predominantly from the right side (65.1%), followed by the left (34.0%), with one midline approach (0.9%). Wrapping of the aneurysm was required in 3 cases (2.8%). Detailed information on the intraoperative characteristics is summarized in Table 2.

### 3.2. Main Target Criteria

Complete aneurysm occlusion was confirmed by the surgeon intraoperatively in 101 patients (97.1%). Angiographic control demonstrated complete occlusion in 94 patients (92.2%), with no residual necks or aneurysmal remnants (see Table 2).

Revision surgery was required in 4 patients (3.8%) and postoperative bleeding occurred in 6 patients (5.7%). Cerebral infarction occurred in 9 (8.5%), new neurological deficits (NND) developed in 18 patients (17.0%), of which 12 were transient (11.3%) and 6 permanent (5.7%). Postoperative epileptic seizures occurred in 7 patients (6.60%). Three patients died during the postoperative course, resulting in an overall mortality of 2.8%. One death was directly related to surgery (fulminant postoperative hemorrhage), while the other two resulted from medical complications unrelated to surgery (heart failure and pulmonary embolism). Notably, univariate analysis, posterior fundus projection showed a higher rate of permanent new neurological deficits (pNND) compared with non-posterior aneurysm locations (20% vs. 4%; OR 5.8, 95% CI 0.9–36.3; Fisher’s exact *p* = 0.09). Although this difference did not reach statistical significance, the magnitude of the effect is notable. Detailed information on the postoperative outcome is summarized in Table 3.

### 3.3. Logistic Regression Models

Logistic regression analyses were performed for the defined outcome variables (postoperative tNND, pNND, infarction, and postoperative hemorrhage). Results are presented in Table 4, Table 5, Table 6 and Table 7 for both the full multivariable models and the reduced models obtained through AIC-based stepwise selection. The principal associations identified in the final models are summarized below.

The results have identified a posterior fundus projection as an independent risk factor for postoperative infarction (*p* = 0.028), whereas the use of intraoperative ICG angiography exerted a protective effect (*p* = 0.034). The surgical side of approach (*p* = 0.037) was also protective, while posterior fundus projection (*p* = 0.030) remained an adverse predictor for the development of a pNND.

For tNND, intraoperative ICG angiography (*p* = 0.044) and the right-sided approach (*p* = 0.008) were associated with reduced risk. Postoperative hemorrhage showed significant associations with preoperative ASA (*p* = 0.016), alcohol consumption (*p* = 0.036), and diabetes mellitus (*p* = 0.026).

## 4. Discussion

This retrospective single-center study analyzed 106 patients who underwent microsurgical treatment for AComA aneurysms, placing it among the larger dedicated series focused exclusively on microsurgical clipping of AComA aneurysms. To date, only Nussbaum et al. [34] have reported a substantially larger cohort (n = 300), while other notable registries such as those by Kim et al. [35] (n = 113) and Lai et al. [36] (n = 115) are of comparable size.

The present findings demonstrate a high rate of complete aneurysm occlusion, with intraoperative confirmation by the operating surgeon in 97.1% of cases and angiographically verified complete exclusion in 92.2%. These results reflect the well-established efficacy of microsurgical clipping in achieving durable aneurysm occlusion and are consistent with occlusion rates reported in the existing literature (92.5–98.4%) [34,35,36,37]. Smaller studies (n < 40), including those by Nanda et al. [38] and Moon et al. [39], have reported lower occlusion rates of 81.3% and 90%, respectively. These results underscore the complete occlusion rate achieved in the current cohort, and suggest a potential relationship between caseload and outcome.

Recently published data on endovascular treatment of AComA aneurysms reveals a wide range of occlusion and retreatment rates depending on the device and technique used. Coiling, including stent- and balloon-assisted variants, remains the most frequently employed modality, yet is associated with moderate rates of incomplete occlusion and recurrence. In the meta-analysis by Sattari et al., complete occlusion was achieved in just 77.3% of cases, with retreatment required in 4.2% overall and 7.5% of patients with unruptured aneurysms [23]. These findings are consistent with the results reported by Catapano et al., who observed recurrence and retreatment rates of 13.4% and 9.7%, respectively [40]. The WEB device has emerged as a promising alternative for wide-neck aneurysms, with Adeeb et al. reporting adequate occlusion in 80.6% of treated AComA aneurysms and no major complications [41]. FDs have demonstrated long-term efficacy in selected anterior circulation aneurysms, with Pagiola et al. reporting complete occlusion rates of up to 86.9% in unruptured ACA aneurysms [42]. However, FD use in the AComA region remains controversial due to the risk of perforator infarction and delayed rupture. Li et al. emphasized that aneurysms smaller than 4 mm carry a fivefold increased risk of intraoperative rupture during coiling, and highlighted the importance of careful device selection in this anatomically sensitive region [25]. Our findings demonstrate a strong durability signal for microsurgical clipping, reflected by a retreatment rate of only 0.9%, which aligns with prior microsurgical series and contrasts with higher retreatment rates reported for endovascular techniques.

Postoperative complications were infrequent. Postoperative new neurological deficits were observed in 18 patients, with only six cases (5.7%) resulting in permanent impairment. Logistic regression analysis demonstrated a significant association between posterior aneurysm projection and both infarction and pNND. These findings highlight the critical role of aneurysm projection in surgical planning and risk stratification. Posteriorly projecting aneurysms, due to their anatomical proximity to perforating arteries and the hypothalamic region, pose a higher risk for ischemic complications—likely attributable to limited intraoperative visualisation and increased manipulation of adjacent perforators. Consequently, meticulous dissection and precise clip placement are essential [33]. Moreover, aneurysm projection should be considered in the decision-making process when choosing between endovascular and microsurgical treatment approaches, reinforcing the need for individualized therapeutic strategies based on anatomical configuration, as previously described in several series [32,43,44]. Unlike endovascular procedures, microsurgical clipping in our cohort was performed without perioperative antiplatelet therapy, eliminating medication-related hemorrhagic risks. In contrast, the mandatory dual antiplatelet regimen for several endovascular techniques introduces additional thromboembolic and bleeding complications.

Intraoperative ICG angiography was employed in 41 of the 106 procedures (38.7%) and intraoperative neuromonitoring in 31 procedures (29.3%). Intraoperative ICG angiography was first introduced into our surgical routine in 2011 with occasional use, and from 2016 onward, both intraoperative ICG angiography and intraoperative Neuromonitoring were implemented as standard modalities in every case. The data suggest a remarkable protective effect of intraoperative ICG angiography, particularly with regard to reducing postoperative infarctions and thereby lowering the risk of tNND. These findings highlight the role of intraoperative ICG angiography as an important adjunct in microsurgical aneurysm treatment, improving both surgical safety and patient outcomes, and are consistent with prior reports demonstrating reduced vascular injury–related morbidity with intraoperative ICG angiography [45,46]. In contrast, neuromonitoring with SSEPs and MEPs did not show a significant association with outcome parameters in bivariate or regression analyses. This may reflect limitations in sensitivity, as previously reported in the literature, where the detection of ischemic events by neuromonitoring has been shown to vary considerably depending on monitoring protocols and interpretation thresholds [47]. Notably, in this subgroup, only one case of pNND due to infarction was observed in 41 aneurysm clippings, highlighting the synergistic potential of integrating both modalities. This stepwise integration likely contributed to the continuous improvement of outcomes over time. In addition, advances in surgical skill, refinements in microsurgical technique, preoperative imaging, and the integration of hybrid operating theatres with high-resolution intraoperative angiography further strengthened perioperative safety.

Taken together, our results corroborate recent reports demonstrating that the combined use of intraoperative ICG angiography and intraoperative neuromonitoring enhances procedural safety in aneurysm surgery, while also reflecting the high procedural safety and favorable outcomes documented in contemporary microsurgical benchmark series [48].

## 5. Limitations

This study has several limitations that must be acknowledged. The retrospective design inherently introduces potential biases, including incomplete data capture, as noted in the table descriptions, and variability in documentation quality. Although experienced cerebrovascular neurosurgeons performed all surgical procedures and the indication for clipping was set in an interdisciplinary cerebrovascular board, the single-center nature of the study limits generalizability and may reflect institutional preferences in treatment selection and intraoperative technique.

Furthermore, the cohort likely represents a selected subset of AComA aneurysms—those deemed suitable for microsurgical clipping by an interdisciplinary cerebrovascular board. As such, aneurysms with favorable anatomy for endovascular treatment may have been excluded, potentially skewing the dataset toward more complex cases.

The long observation period spanning nearly two decades introduces heterogeneity in surgical technique, imaging modalities, and perioperative management. While this reflects real-world clinical evolution, it may confound outcome comparisons across time, as the period also encompasses significant advances in both microsurgical and endovascular techniques. No subgroup analysis by time period was performed, which may limit interpretation of temporal trends.

Frequent comparisons with endovascular results from the literature may involve non-contemporaneous comparison bias, which should be considered when interpreting our findings.

Neurocognitive outcomes and quality-of-life measures were not systematically assessed, although these parameters are increasingly recognized as essential endpoints in aneurysm treatment.

## 6. Conclusions

Our study demonstrates that microsurgical clipping of unruptured AComA aneurysms is a highly effective treatment modality in selected cases, achieving high rates of complete angiographic occlusion and low rates of permanent neurological deficits. The data suggest that aneurysm projection significantly influences postoperative outcomes, with posteriorly projected aneurysms associated with increased risk of infarction and neurological complications. Accordingly, aneurysm projection should be considered when selecting the optimal treatment strategy, whether endovascular or microsurgical. Intraoperative ICG angiography proved beneficial in reducing ischemic events and may be recommended as an adjunct intraoperative tool.

Despite the increasing availability of endovascular techniques, microsurgical clipping remains a valuable option, particularly in cases with complex anatomy, wide necks, or prior endovascular failure. These findings support the continued role of microsurgery in the interdisciplinary management of AComA aneurysms and highlight the importance of individualized treatment planning based on anatomical and radiological characteristics.

Future prospective studies are needed to validate these findings and further refine interdisciplinary treatment strategies for AComA aneurysms.

## Figures and Tables

**Table 1:** Descriptive statistics of preoperative patient- and aneurysm-specific parameters. For categorial variables, absolute frequencies (percent) are reported without missing values. ADPKD = autosomal dominant polycystic kidney disease, SAH = subarachnoidal haemorrhage, max. = maximal, mm = millimeter, prev. = previously. ASA = American Society of Anesthesiologists. ^a^ = data missing for 4 patients, ^b^ = data missing for 26 patients, ^c^ = data missing for 13 patients, ^d^ = data missing for 10 patients, ^e^ = data missing for 2 patients, ^f^ = data missing for 8 patients.

Preoperative Parameters		Value
Number of cases (n)		106
**Patient-specific parameters**		
Mean Age in years (standard deviation)		56.42 (10.27)
ADPKD ^a^		3 (2.94%)
Mean stay in hospital in days (standard deviation)		18.48 (16.33)
SAH in anamnesis		12 (11.3%)
Distribution of ASA Score ^b^		
1		12 (15.00%)
2		47 (58.75%)
3		20 (25.00%)
4		0 (0%)
5		1 (1.25%)
Arterial hypertension ^c^		59 (63.44%)
Smoking ^d^		41(42.71%)
Diabetes Mellitus ^e^		4 (3.85%)
Alcohol ^f^		16 (16.33%)
**Aneurysm-specific parameters**		
Fundus Projection	anterior	50 (47.17%)
	superior	32 (30.19%)
	inferior	14 (13.21%)
	posterior	10 (9.43%)
Max. Diameter mean in mm (range)		5.92 (1.00–19.00)
Prev. Coiled	yes	7 (6.60%)
Thrombosed	yes	1 (0.94%)
Blebs	yes	60 (56.60%)
Calcifications	yes	5 (4.72%)
Number of other aneurysms	0	68 (64.15%)
	1	30 (28.30%)
	2	7 (6.60%)
	3	1 (0.94%)

**Table 2:** Intraoperative parameters. For categorical variables, absolute frequencies (percent of non-missing values) are reported. MEP = motor evoked potentials, SSEP = somatosensory evoked potentials, ICG = indocyanine green angiography.

Intraoperative Parameters		Value (%)
Reposition of clips		21 (19.81%)
Average number of clips		1.35 (0–3)
Number of Clips	1	71 (66.98%)
	2	30 (28.30%)
	3	4 (3.77%)
Intraoperative Rupture of aneurysm		3 (2.83%)
Temporary Clipping		12 (11.32%)
Intraoperative Neuromonitoring (MEP, SSEP)		31 (29.25%)
Intraoperative ICG angiography		41(38.68%)
Side of approach	right	69 (65.09%)
	left	36 (33.96%)
	median	1 (0.94%)
Wrapping		3 (2.83%)
Main Target Criteria		
	Complete occlusion (surgeon)	101 (97.12%)
	Complete occlusion (angiographic)	94 (92.16%)

**Table 3:** Postoperative complications. For categorical variables, absolute frequencies (percent of non-missing values) are reported. cSDH = chronic subdural hematoma.

Postoperative Complications	Value (%)
Revision surgery	4 (3.77%)
Postoperative hemorrhage	6 (5.66%)
Remote bleeding	2 (1.89%)
cSDH	0 (0%)
Infarction	9 (8.49%)
Neurological deficit	18 (16.98%)
Permanent deficit	6 (5.66%)
Transient deficit	12 (11.32%)
Postoperative seizure	7 (6.60%)
Surgical site infection	13 (12.26%)
Perioperative death	3 (2.83%)
Recurrence	1 (0.94%)
Retreatment rate	1 (0.94%)

**Table 4:** This table reports the results of the logistic regression model for the outcome parameter tNND. tNND = transient new neurological deficit. Stepwise variable selection was performed using the Akaike Information Criterion (AIC); ADPKD = autosomal dominant polycystic kidney disease, ICG = indocyanine green angiography, SAH = subarachnoidal hemorrhage, IONM = intraoperative neuromonitoring, AcomA = Arteria communicans anterior aneurysm, mASA = American Society of Anesthesiologists classification.

	Estimate	ci-Lower	ci-Upper	*p*-Value
**Penalized likelihood logistic regression model für tNND**				
Intercept	−0.455	−1.951	0.939	0.523
Superior Projection	0.266	−1.265	1.778	0.726
Posterior Projection	−0.396	−5.332	2.112	0.792
Inferior Projection	1.009	−0.726	2.732	0.243
Diabetes	0.023	−0.168	0.198	0.798
Calcification	0.379	−4.657	3.116	0.823
Blebs	−0.939	−2.265	0.297	0.136
Intraoperative Neuromonitoring	0.383	2.394	5.410	0.809
Intraoperative ICG angiography	−1.413	−6.414	1.005	0.290
Side of approach (right)	−1.692	−3.161	−0.411	0.009
Clip Repositioning	0.839	−0.789	2.456	0.300
**Penalized likelihood logistic regression model for transient deficit after variable selection**				
Intercept	−0.066	−1.201	1.049	0.906
Diabetes	0.039	−0.134	0.193	0.634
Blebs	−1.171	−2.532	0.066	0.064
Intraoperative ICG angiography	−1.418	−3.161	−0.038	0.044
Right-sided approach	−1.663	−3.032	−0.424	0.008

**Table 5:** This table reports the results of the logistic regression model for the outcome parameter pNND. pNND = permanent new neurological deficit. Stepwise variable selection was performed using the Akaike Information Criterion (AIC); ADPKD = autosomal dominant polycystic kidney disease, ICG = indocyanine green angiography, SAH = subarachnoidal hemorrhage, IONM = intraoperative monitoring, AcomA = Arteria communicans anterior aneurysm, mASA = American Society of Anesthesiologists classification.

	Estimate	ci-Lower	ci-Upper	*p*-Value
**Penalized likelihood regression model for pNND**				
Intercept	−1.926	−4.362	−0.182	0.029
Superior Projection	0.961	−1.184	3.446	0.374
Posterior Projection	2.611	0.226	5.604	0.032
Inferior Projection	1.311	−1.291	3.905	0.292
Diabetes	−0.117	−0.509	0.132	0.389
Calcification	1.586	−3.448	4.815	0.416
Blebs	−0.343	−2.115	1.336	0.681
IONM	0.935	−2.282	6.079	0.587
Intraoperative ICG angiography	−1.933	−7.137	0.943	0.222
Side of surgical approach (right)	−1.873	−4.069	−0.118	0.036
Clip Repositioning	1.348	−1.114	3.702	0.252
**Penalized likelihood regression model for pNND after manual backward selection**				
Intercept	−2.168	−4.438	−0.670	0.003
Superior Projection	1.011	−1.108	3.482	0.344
Posterior Projection	2.481	0.257	5.111	0.030
Inferior Projection	1.394	−1.229	4.041	0.270
Intraoperative ICG angiography	−1.272	−3.724	0.528	0.177
Side of surgical approach (right)	−1.763	−3.735	−0.104	0.037

**Table 6:** This table reports the results of the logistic regression model for the outcome parameter postoperative Infarction. Stepwise variable selection was performed using the Akaike Information Criterion (AIC); ADPKD = autosomal dominant polycystic kidney disease, ICG = indocyanine green angiography, SAH = subarachnoidal hemorrhage, IONM = intraoperative monitoring, AcomA = Arteria communicans anterior aneurysm, mASA = American Society of Anesthesiologists classification.

	Estimate	ci-Lower	ci-Upper	*p*-Value
**Penalized-likelihood logistic regression model for infarct**				
Intercept	−1.784	−4.135	−0.104	0.036
Superior Projection	1.660	−0.247	4.133	0.090
Posterior Projection	3.055	0.606	6.139	0.015
Inferior Projection	1.859	−0.337	4.400	0.095
Diabetes	−0.076	−0.336	0.136	0.494
Calcification	1.560	−3.476	4.740	0.421
blebs	−1.173	−2.957	0.295	0.120
IONM	0.898	−2.367	6.209	0.613
Intraoperative ICG angiography	−2.341	−7.666	0.432	0.112
Side of approach (right)	−1.589	−3.356	−0.044	0.044
Clip Repositioning	1.519	−0.484	3.533	0.130
**Penalized-likelihood logistic regression model for infarct after manual backward selection**				
Intercept	−1.477	−3.776	0.136	0.075
Superior Projection	1.360	−0.488	3.753	0.154
Posterior Projection	2.534	0.283	5.204	0.028
Inferior Projection	1.726	−0.491	4.290	0.124
Diabetes	−0.052	−0.291	0.150	0.628
Blebs	−1.127	−2.908	0.353	0.139
Intraop ICG Angiography	−1.882	−4.452	−0.129	0.034
Side of approach (right)	−1.264	−2.773	0.157	0.081

**Table 7:** This table reports the results of the logistic regression model for the outcome parameter postoperative bleeding. Stepwise variable selection was performed using the Akaike Information Criterion (AIC); ADPKD = autosomal dominant polycystic kidney disease, ICG = indocyanine green angiography, SAH = subarachnoid hemorrhage, IONM = intraoperative monitoring, AcomA = Arteria communicans anterior aneurysm, ASA = American Society of Anesthesiologists classification.

	Estimate	ci-Lower	ci-Upper	*p*-Value
**Penalized likelihood regression model for postoperative bleeding**				
Intercept	−2.784	−13.476	1.716	0.224
mASA	1.088	−0.465	4.455	0.181
Arterial Hypertension	1.192	−1.046	6.158	0.293
ADPKD	2.095	−4.531	10.269	0.429
Diabetes	−0.015	−5.508	6.567	0.995
Nicotine Abuse	−0.127	−2.113	3.446	0.899
Alcohol consumption	0.986	−2.509	4.387	0.451
Previously Coiled AcomA	−0.588	−5.751	4.994	0.765
SAH	0.395	−5.656	4.508	0.835
Other Aneurysms	−1.579	−16.298	6.749	0.665
Number of other aneurysms	1.670	−5.952	12.755	0.571
factor preoperative imaging	−0.190	−8.892	3.703	0.918
factor superior projection	−0.176	−3.231	3.079	0.882
factor posterior projection	−0.536	−6.048	3.937	0.766
factor inferior projection	−2.996	−12.021	2.582	0.319
Diabetes	0.196	−0.196	0.738	0.242
Calcification	1.227	−5.031	8.502	0.661
Blebs	−0.162	−2.449	2.985	0.883
IONM	−2.533	−9.761	3.017	0.307
Intraoperative ICG angiography	2.622	−3.281	10.481	0.315
Side of approach right	−1.062	−3.708	0.889	0.288
Number of Clips used	0.543	−1.251	3.874	0.51
Clip Repositioning	−0.677	−4.192	1.784	0.6
Temporal Clipping	−2.337	−7.685	0.723	0.148
**Penalized likelihood regression model for postoperative bleeding after manual backward selection**				
Intercept	−3.325	−7.050	−1.540	<0.001
mASA	2.181	0.338	5.591	0.016
Alcohol consumption	3.032	0.193	7.917	0.036
Diabetes	0.458	0.052	1.234	0.026
Side of approach (right)	−2.180	−6.510	0.440	0.107
Temporal Clipping	−2.956	−9.295	0.475	0.104

## Data Availability

The original contributions presented in this study are included in the article. Further inquiries can be directed to the corresponding author.

## Abbreviations

The following abbreviations are used in this manuscript:

AComA	Anterior communicating artery
ADPKD	Autosomal dominant polycystic kidney disease
ASA	American Society of Anesthesiologists
cSDH	chronic subdural hematoma
CTA	Computed Tomography Angiography
DSA	Digital Subtraction Angiography
FD	Flow Diverter
ICG	Indocyanine green angiography
ICH	Intracerebral hemorrhage
MEP	Motor evoked potential
MRA	Magnetic Resonance Angiography
NND	New neurological deficit
pNND	Permanent new neurological deficit
RROC	Raymond–Roy occlusion classification
SAH	Subarachnoid hemorrhage
SD	Standard deviation (SD)
SSEP	Somatosensory evoked potential
SSI	Surgical site infection
tNND	Transient new neurological deficit
UIA	Unruptured intracranial aneurysm
WEB	Woven Endo Bridge
